# Resource Gain and Cross-Domain Spillover: How Work Connectivity Behavior After Hours Sustains Work Engagement

**DOI:** 10.3390/bs16020254

**Published:** 2026-02-10

**Authors:** Guangli Lu, Yingfei Li, Jinfeng Li, Chaoran Chen

**Affiliations:** 1 School of Business, Henan University, Jinming Avenue, Kaifeng 475004, China; 10090010@vip.henu.edu.cn (G.L.); ljinf@henu.edu.cn (J.L.); 2 Institute of Nursing and Health, School of Nursing and Health, Henan University, Jinming Avenue, Kaifeng 475004, China; kfccr@henu.edu.cn

**Keywords:** work connectivity behavior after-hours, thriving at work, work-family enrichment, workplace mindfulness, work engagement

## Abstract

With the widespread adoption of information technology, work connectivity behavior after hours has become a common phenomenon in organizations, yet its potential positive effects on employees remain to be explored. Guided by Conservation of Resources theory and integrating the Job Demands-Resources model with the Work-Home Resources model, this study integrated work and family domains to investigate how work connectivity behavior after hours influences work engagement from a resource perspective. Analysis of data from 327 Chinese employees revealed a positive association between work connectivity behavior after hours and work engagement. This relationship was found to be mediated by employees’ thriving at work and their experience of work-family enrichment. Furthermore, workplace mindfulness was found to moderate the association between work connectivity behavior after hours and thriving at work, as well as the strength of the overall indirect pathway to work engagement. These findings not only extend the current literature on work connectivity behavior after hours but also provide novel perspectives for organizations to effectively manage such connectivity beyond working hours.

## 1. Introduction

With the development of the mobile internet economy and the enhancement of remote communication tools, many employees unconsciously attend to work-related information during non-work time and even consider the handling of work tasks as a habit or responsibility (Schlachter et al., 2018). The behavior of employees using portable electronic devices to engage in work or communicate with colleagues during non-work time has become a common phenomenon (van Zoonen et al., 2023), which is termed work connectivity behavior after hours (WCBA) (Richardson & Benbunan-Fich, 2011). Existing research on WCBA has extensively revealed its negative effects and underlying mechanisms. For example, Xu et al. (2022) demonstrated that WCBA facilitates employee time theft through the mechanisms of emotional exhaustion and moral disengagement. Y. Liu et al. (2024) revealed that WCBA enhances employees’ unethical pro-family behavior from work-family perspectives. However, within the context of accelerating digitalization and widespread flexible work arrangements, WCBA may positively influence employees’ work attitudes and behaviors through specific mechanisms. Several studies have indicated that WCBA exerts a dual influence on employees’ occupational mental health (Zhu et al., 2024), creativity (Yuan et al., 2023), and other work-related attitudes and behaviors. This suggests that WCBA is not exclusively detrimental and its positive effects have also attracted increasing scholarly attention. Notwithstanding this evidence, its potential positive effects and underlying mechanisms have not been fully explored in existing studies (Shi et al., 2018).

Work engagement, defined as a positive, fulfilling, and work-related psychological state (Schaufeli et al., 2006), is a salient manifestation of employees’ attitudes and behaviors at work (Timms et al., 2015). Employees with high work engagement play a pivotal role in driving organizational prosperity. WCBA changes employees’ work patterns and schedules, potentially impacting work engagement. Yang et al. (2023) revealed the positive impact of WCBA on work engagement and found that, within the context of employees working from home, WCBA positively influences work engagement by increasing work autonomy and reducing emotional exhaustion. Zhang et al.’s (2023) diary study of public hospital doctors revealed that the use of work-related information and communication technology after hours exhibits an inverted U-shaped relationship with next day work engagement. These studies have preliminarily explored how WCBA relates to work engagement within the work domain, but their differing findings indicate that further research is necessary. In the post-pandemic era, as remote and hybrid work arrangements become normalized, WCBA, as a behavior that blurs the boundaries between work and family, inevitably influences both the work and family domains. Specifically, WCBA may not only influence employees’ psychological resources and functional states in the work domain, but also their performance in the family domain through the cross-domain transfer and transformation of resources. Meanwhile, support and a sense of accomplishment from the family domain can generate positive feedback, infusing employees’ work with renewed motivation, thereby forming a dynamic cycle of resources that spans both work and family domains. To further understand the relationship between WCBA and work engagement, this study selects the more positive constructs of thriving at work and work-family enrichment as mediating variables, integrating the work and family domains to examine the impact of WCBA.

Additionally, considering that WCBA’s inherent stressor attributes could undermine the realization of these beneficial pathways, it is necessary to identify boundary conditions that can simultaneously mitigate stress risks and reinforce gain mechanisms. Mindfulness is a state of consciousness or an individual trait, reflecting individual differences in awareness, attention, and nonjudgmental acceptance (Brown et al., 2007). Studies have shown that mindfulness at work can serve as a critical personal resource, helping individuals manage stress, regulate emotions, and maintain cognitive focus (Hülsheger et al., 2013; Tong et al., 2021). Consequently, workplace mindfulness may function as a crucial boundary condition. Concurrently, workplace mindfulness is a malleable trait that is capable of being cultivated and developed through mindfulness training (Zheng et al., 2023). This provides viable opportunities for organizational practice.

Therefore, guided by Conservation of Resources (COR) theory (Hobfoll, 1989) and integrating the Job Demands-Resources (JD-R) model (Bakker & Demerouti, 2007) with the Work-Home Resources (W-HR) model (Ten Brummelhuis & Bakker, 2012), this study constructs a parallel mediation model to investigate the mediating roles of thriving at work and work-family enrichment from a resource perspective, while exploring the moderating role of workplace mindfulness. While prior research has confirmed a significant link between WCBA and work engagement, this study proposes a novel mediating model. This model not only encompasses both the work and family domains but also incorporates cross-domain resource dynamics, which have not yet been fully explored in the existing literature. Furthermore, as previously highlighted, existing research on WCBA has mainly focused on its resource-depleting effects. In contrast, this study expands the current discourse by shifting the attention to positive outcomes, and explains how WCBA positively affects work engagement from the angle of resource gain and spillover, providing a new perspective for understanding WCBA.

## 2. Theory and Hypotheses

COR theory provides a theoretical framework from a resource perspective. It posits that individuals have an inherent tendency to acquire, retain, cultivate, and protect valued resources (Hobfoll, 1989). Job resources provide employees with material and psychological support, facilitating their learning, growth, and development, reducing the effort or costs required to meet job demands, and fostering work engagement through positive gain spirals (Hakanen et al., 2008). While COR effectively explains the motivation to acquire and protect resources, it alone is insufficient to fully explicate the cross-domain resource pathways central to our model. To address these gaps and provide a more nuanced explanation, we incorporate the JD-R model and the W-HR model into our framework.

According to the JD-R model, job resources are viewed as supportive factors that facilitate task completion, alleviate psychological stress, and stimulate personal development (Demerouti et al., 2001). Such resources not only enhance employee performance but also buffer against the adverse effects of job demands (Bakker & Demerouti, 2007). In this context, WCBA can be considered a job resource, as it supports individuals in completing work tasks (Zhang et al., 2023). The JD-R model thus allows us to hypothesize that WCBA may directly promote work engagement and buffer the impact of demands by activating internal growth dynamics and reducing strain. Furthermore, WCBA may act as a pathway for acquiring other job resources, such as increased work autonomy (van Zoonen et al., 2023), which helps satisfy basic psychological needs. However, the primary focus of the JD-R model remains within the boundary of the work domain.

Given that WCBA inherently blurs the work-family boundary, a complete understanding of its impact requires a theory that explicitly accounts for cross-domain resource dynamics. The W-HR model extends the resource perspective by proposing that resources generated in one domain can spill over or transfer to the other domain, promoting positive outcomes or buffering stressors (Ten Brummelhuis & Bakker, 2012). Specifically, resources acquired through work can enhance family functioning and satisfaction by extending into the family domain. Concurrently, abundant family resources reciprocally strengthen the work domain by boosting job effectiveness and resource acquisition capabilities, forming a virtuous cycle. Applying this lens, WCBA as a job resource enables individuals to build up resources at work, which can then benefit their family roles by spilling over into the family domain (He et al., 2023). Concurrently, resources from the family domain can also positively spill over into the work domain. These resources, such as support, positive emotions, and restored energy, enhance employees’ capacity and willingness to utilize WCBA effectively.

### 2.1. Work Connectivity Behavior After Hours and Work Engagement

The JD-R model proposes that job resources foster motivation and positive outcomes through an energizing pathway (Bakker & Demerouti, 2017). As a job resource that transcends traditional workplace boundaries, WCBA not only meets immediate task requirements but also provides employees with continuous access to social, informational, and instrumental support during non-work time. WCBA enables employees to perform their tasks at flexible times, thereby allowing them to maintain higher energy levels and achieve deeper engagement in their work (Zhang et al., 2023). Concurrently, employees can readily acquire task-related guidance, collaborative opportunities, and real-time feedback through this connectivity. When employees address work during non-work time, the experience of skill development, problem-solving accomplishment, or heightened job control may catalyze work engagement.

Furthermore, as a pathway to acquiring job resources, WCBA facilitates resource gains that enhance work engagement through fulfilling employees’ basic psychological needs or facilitating goal attainment (Shi et al., 2018). On the one hand, WCBA breaks down traditional spatiotemporal boundaries of work, providing employees with greater autonomy, flexibility, and social connectedness. The fulfillment of employees’ autonomy needs can lead to an increase in their work-related psychological resources (van Zoonen et al., 2023), which promotes proactive behaviors (He et al., 2023). On the other hand, WCBA increases the likelihood of employees simultaneously juggling work and family roles. Resource gains from the work domain exert a positive spillover effect on the family domain, thus correspondingly promoting work-family enrichment (Yang et al., 2022). This cross-domain enrichment catalyzes higher work engagement through reciprocal resource dynamics. The following hypothesis is therefore proposed:

**H1.**  *Work connectivity behavior after hours is positively related to work engagement.*

### 2.2. The Mediating Role of Thriving at Work

Thriving at work refers to a psychological state characterized by individuals’ simultaneous experience of vitality and learning in their work. Employees who thrive at work can engage in continuous self-growth and development, which helps enhance organizational effectiveness and fosters organizational prosperity (D. Liu et al., 2021).

As a new work scenario, WCBA transcends temporal and spatial boundaries, promoting greater autonomy and control among employees (Yang et al., 2023). Concurrently, WCBA provides employees and the organization with the capability for real-time connection, which enhances communication and feedback efficiency and strengthens information sharing within the organization (Yang et al., 2022). When employees work in an environment that promotes decision-making discretion, extensive information sharing, and a climate of trust and respect, they are more likely to achieve thriving (D. Liu et al., 2021). According to the JD-R model, thriving at work can be considered as a personal resource. The accumulation of this resource helps employees cope with work-related stress and enhances engagement (Porath et al., 2012). On the one hand, the vitality inherent in thriving directly fuels the vigor dimension of engagement, providing employees with the sustained psychological energy necessary to invest significant effort and persist in the face of challenges. On the other hand, the mastery and growth associated with learning facilitate deeper cognitive immersion in work activities, enabling employees to derive greater meaning and purpose from their tasks, thereby deepening their dedication. Employees who thrive at work are more likely to perceive their work as meaningful and valuable, thereby exhibiting heightened work engagement (Porath et al., 2012). The following hypothesis is therefore proposed:

**H2.**  *Thriving at work mediates the positive relationship between WCBA and work engagement.*

### 2.3. The Mediating Role of Work-Family Enrichment

Work-family enrichment describes the degree to which engagement in one role improves an individual’s functioning and well-being in another role (Greenhaus & Powell, 2006), including both enrichment from work to family and from family to work.

As a job resource that reduces disruptions and supports the achievement of work goals, WCBA enables employees to better integrate work and life domains (He et al., 2023). The sense of accomplishment and satisfaction derived from the work domain may positively spill over into family life. Work-to-family enrichment occurs when resources acquired in the workplace enrich employees’ personal resources and are subsequently applied to enhance outcomes in the family domain (Carlson et al., 2019). Concurrently, skills and knowledge gained from WCBA can be applied to managing challenges within the family domain, which helps to gain family support. Family support provides resources that enhance work capabilities and performance, fostering family-to-work enrichment (McNall et al., 2010). With enriched family support, employees demonstrate greater motivation to acquire resources and seek opportunities to obtain resources (Greenhaus & Powell, 2006). Employees with work-family enrichment experience often view connectivity behaviors as a chance for resource gain, thus increasing their work engagement. The following hypothesis is therefore proposed:

**H3.**  *Work-family enrichment mediates the positive relationship between WCBA and work engagement.*

### 2.4. The Moderating Role of Workplace Mindfulness

Workplace mindfulness is defined as an individual’s awareness and attention to their present internal experiences and external work environment, with an attitude of acceptance (Zheng et al., 2023). Employees with varying levels of workplace mindfulness may exhibit distinct behavioral patterns. Those with higher workplace mindfulness may process task demands during non-work time more efficiently, reduce frustration caused by time encroachment, and diminish perceived threats of boundary permeability through cognitive reappraisal. They tend to approach workplace challenges with greater positivity (Zivnuska et al., 2016). When faced with WCBA, employees with high workplace mindfulness are more effective at utilizing job resources during non-work time to complete tasks and balance work and life domains. The following hypotheses are therefore proposed:

**H4.**  *Workplace mindfulness positively moderates the relationship between WCBA and thriving at work.*

**H5.**  *Workplace mindfulness positively moderates the relationship between WCBA and work-family enrichment.*

According to the JD-R model, personal resources can explain the mechanism through which various job resources translate into work engagement (Xanthopoulou et al., 2007). Concurrently, according to the W-HR model, personal resources can help employees effectively leverage the transformation of contextual resources and the management of contextual demands between work and family domains, facilitate the maintenance and growth of resource reserves, and then benefit outcomes in the corresponding domain (Ten Brummelhuis & Bakker, 2012). As a personal resource, workplace mindfulness may influence employees’ perception of job characteristics and their coping strategies, thus moderating the strength of the paths. Therefore, this research develops a moderated mediation model. Specifically, when employees exhibit a high level of workplace mindfulness, the positive impact of WCBA on work engagement through thriving at work and work-family enrichment is strengthened. The following hypotheses are therefore proposed:

**H6.**  *Workplace mindfulness moderates the indirect effect of work connectivity behavior after hours on work engagement through thriving at work.*

**H7.**  *Workplace mindfulness moderates the indirect effect of work connectivity behavior after hours on work engagement through work-family enrichment.*

**Figure 1:**
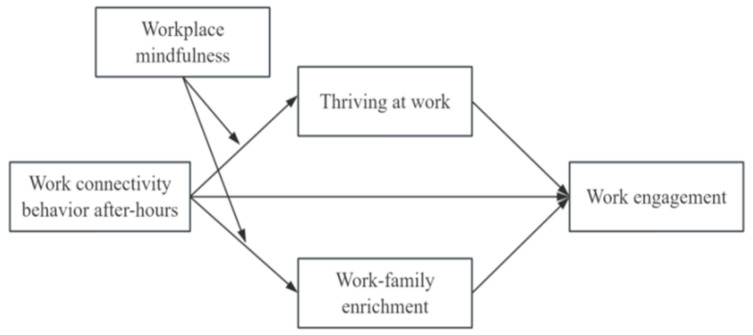
Theoretical model.

Figure 1 presents the hypothesized framework for the research.

## 3. Method

### 3.1. Sample and Data Collection

Before collection, the sample size was determined by considering both statistical power requirements and practical survey constraints. Specifically, we employed G*Power 3.1 software (Düsseldorf, Germany) to conduct an a priori power analysis. Guided by Ferguson’s (2009) recommendation for a minimum meaningful effect size in social science research, we set the effect size at 0.04 for this analysis. Using the linear multiple regression test, with an alpha level of 0.05 and statistical power set at 0.9, the analysis indicated that a minimum sample size of 265 was required. Considering the potential for invalid responses and attrition during the questionnaire collection process, we planned to distribute at least 400 questionnaires in the first phase. Data were subsequently collected through the online survey platform Credamo (Beijing, China). Participants were required to be full-time employees working in organizations where WCBA was common or permitted. To reduce common method bias, the questionnaire was administered in three waves, with small monetary rewards provided to participants who completed all survey waves and satisfied the data quality standards. Specifically, during the first phase (8 November 2024), data on WCBA and workplace mindfulness were collected alongside participants’ basic demographic information, including gender, age, and education. The second phase (23 November 2024) measured participants’ thriving at work and work-family enrichment. The third phase (8 December 2024) measured participants’ work engagement. Ultimately, we successfully collected 377 initial questionnaires. Participants were drawn from multiple industries, including manufacturing, finance, internet services, public institutions and other fields. After screening, 327 valid responses were retained for analysis. This final sample size exceeds the minimum required as determined by our a priori power calculation, thereby ensuring sufficient statistical power for this study.

The sample demographics are summarized as follows. The gender composition of the sample was represented by 182 females and 145 males. The age distribution of the participants showed that the 26–35 age bracket was the largest, making up 52.6% of the total. Meanwhile, participants under 25, in the 36–45 range, and over 46 accounted for approximately 19%, 21.1%, and 7.3% of the sample, respectively. In terms of education, approximately 4% had a high school education or less, 6.7% had specialist education, 62.1% had a bachelor’s degree, and 27.2% had a master’s degree or higher. The percentages of married and unmarried participants were 56.6% and 43.4%, respectively. Regarding work experience, approximately 12.2% of participants had under one year, 14.1% had 2–3 years, 14.7% had 4–5 years, 22.3% had 6–10 years, and 36.7% had over 10 years.

### 3.2. Measures

This study employed widely adopted scales. All measures used a 5-point Likert scale, with those lacking official Chinese versions being translated through back-translation.

Work connectivity behavior after hours was assessed with the scale developed by Richardson and Thompson (2012) and subsequently adapted into Chinese by Wu et al. (2018). The scale consisted of two dimensions: frequency and duration, with a total of 7 items (4 items for frequency, 3 items for duration). Frequency items range from 1, “Never”, to 5, “Always”, and duration items range from 1, “0–15 min”, to 5, “Over 2 h”. In this study, the Cronbach’s alpha was 0.862.

Thriving at work was assessed with the scale developed by Porath et al. (2012) and subsequently adapted into Chinese by Han and Wei (2013). The scale consisted of two dimensions: learning and vitality, each containing 5 items, totaling 10 items. A sample is “I find myself learning often” (1 = “strongly disagree”, 5 = “strongly agree”). In this study, the Cronbach’s alpha was 0.738.

Work-family enrichment was assessed with the scale developed by Carlson et al. (2006) and subsequently adapted into Chinese by Tang et al. (2009). The scale consisted of two dimensions: work-to-family enrichment and family-to-work enrichment, each containing 7 items, totaling 14 items. A sample is “Working helps me master interpersonal skills and improve my communication abilities, enabling me to play a more successful role in my family” (1 = “strongly disagree”, 5 = “strongly agree”). In this study, the Cronbach’s alpha was 0.958.

Workplace mindfulness was assessed with the scale developed by Zheng et al. (2023). The scale consisted of three dimensions: awareness, attention, and acceptance, each containing 6 items, totaling 18 items. A sample is “I can stay aware of my feelings when performing work tasks” (1 = “strongly disagree”, 5 = “strongly agree”). In this study, the Cronbach’s alpha was 0.919.

Work engagement was assessed with the short version of Schaufeli et al.’s (2006) scale. The scale consisted of three dimensions: vigor, dedication, and absorption, each containing 3 items, totaling 9 items. A sample is “I am enthusiastic about my job” (1 = “strongly disagree”, 5 = “strongly agree”). In this study, the Cronbach’s alpha was 0.925.

To account for potential confounding influences, this study controlled for employees’ marital status (Minnotte et al., 2010), along with other key demographics including gender, age, education, and work experience.

## 4. Results

### 4.1. Confirmatory Factor Analysis and Common Method Bias Test

This study employed Mplus8.0 to carry out confirmatory factor analyses, aiming to examine the discriminant validity of the measured variables. Table 1 displays the results. As evidenced by the fit indices (*χ*^2^*/df* = 1.574, RMSEA = 0.042, CFI = 0.905, TLI = 0.901, SRMR = 0.051), the five-factor model fitted the data best compared to the other four models. The results indicated that WCBA, thriving at work, work-family enrichment, workplace mindfulness and work engagement possessed high discriminant validity. Subsequently, common method bias was assessed through Harman’s single-factor test (Podsakoff et al., 2003) using SPSS 22.0 (Armonk, NY, USA). The results showed that the proportion of the first principal component obtained without rotation was 37.58%, which was lower than the critical value of 40% and met the requirements. According to the analysis results in Table 1, the single factor model exhibited significantly poorer model fit (*χ*^2^*/df* = 2.992, RMSEA = 0.078, CFI = 0.669, TLI = 0.657, SRMR = 0.084) compared to the five-factor model. Collectively, these analyses suggested that common method bias remained at acceptable levels and did not substantially threaten the interpretation of results.

### 4.2. Descriptive Statistics and Correlation Analysis

Table 2 summarizes the means, standard deviations, and correlations among all variables. WCBA demonstrated significant positive associations with work engagement, as well as with thriving at work and work-family enrichment, both of which were also positively correlated with work engagement. These findings provide preliminary support for H1, H2, and H3, laying the foundation for subsequent hypothesis testing.

### 4.3. Hypotheses Testing

We employed hierarchical regression analysis in SPSS 22.0 to test the hypotheses. Table 3 displays the results. Specifically, models 1, 4, and 7 display the effects of the control variables on thriving at work, work-family enrichment, and work engagement, respectively. Models 2, 5, and 8 present the regression results of WCBA on these same outcome variables.

#### 4.3.1. Main Effect Test

Results from model 8 confirmed a significant main effect, with WCBA emerging as a positive predictor of work engagement (*β* = 0.221, *p* < 0.001), providing support for H1.

#### 4.3.2. Mediation Effect Test

Results from model 2 demonstrated that WCBA significantly and positively influenced thriving at work (*β* = 0.231, *p* < 0.001). Model 8 indicated that when both WCBA and thriving at work were included in the regression model predicting work engagement, the effect of WCBA remained significant (*β* = 0.077, *p* < 0.05), while thriving at work also showed a significant positive association with work engagement (*β* = 0.622, *p* < 0.001). These results support H2. Similarly, results from model 5 showed that WCBA positively predicted work-family enrichment (*β* = 0.130, *p* < 0.05). In model 10, after including both WCBA and work-family enrichment, the association between WCBA and work engagement remained significant (*β* = 0.135, *p* < 0.001). Concurrently, work-family enrichment positively influenced work engagement (*β* = 0.662, *p* < 0.001), thereby supporting H3.

Further, to assess the indirect effects through thriving at work and work-family enrichment, we conducted mediation analysis following the procedure established by Preacher and Hayes (2008). Specifically, the analysis was conducted using Model 4 of the PROCESS macro, with 5000 bootstrap samples and a 95% confidence interval specified. The results in Table 4 confirmed a significant indirect effect of WCBA on work engagement through thriving at work (effect = 0.061, 95% CI [0.026, 0.108]), as the confidence interval did not include zero, thus fully supporting H2. Similarly, the indirect effect of WCBA on work engagement through work-family enrichment was significant (effect = 0.055, 95% CI [0.008, 0.109]), as the confidence interval also excluded zero. Thus, H3 was fully supported.

#### 4.3.3. Moderating Effects Test

Prior to testing, the independent variable and moderator variable were mean-centered, with their interaction term created to reduce the impact of multicollinearity. In Table 3, model 3 showed that the interaction between WCBA and workplace mindfulness was significantly related to thriving at work (*β* = 0.082, *p* < 0.05), thus H4 was supported. However, the results from model 6 did not support a significant moderating effect of workplace mindfulness on the relationship between WCBA and work-family enrichment (*β* = 0.020, *p* > 0.05). Consequently, H5 was not supported, and H7 was likewise not supported.

#### 4.3.4. Moderated Mediation Effect Test

This study conducted a moderated mediation analysis using the bootstrap method in the PROCESS macro. According to Table 5, the indirect effect of WCBA on work engagement through thriving at work was non-significant under low workplace mindfulness (M − 1 SD, effect = 0.035, 95% CI [−0.043, 0.119]) but was significant under high workplace mindfulness (M + 1 SD, effect = 0.148, 95% CI [0.073, 0.237]). The high-low difference effect coefficient was 0.113 (95% CI [0.004, 0.234]), with the interval also excluding zero. These results provide support for H6 by demonstrating that workplace mindfulness strengthens the indirect effect of WCBA on work engagement through thriving at work.

## 5. Discussion

Based on COR theory, this study explored how WCBA affects employees’ work engagement. First, WCBA was significantly associated with higher work engagement. Second, thriving at work and work-family enrichment mediated the link between WCBA and work engagement. Third, workplace mindfulness positively moderated the association between WCBA and thriving at work and strengthened the mediating effect of thriving at work in the relationship between WCBA and work engagement. However, the relationship between WCBA and work-family enrichment was not moderated by workplace mindfulness. This may be explained by the following considerations. First, workplace mindfulness is particularly concerned with events in the workplace rather than with events occurring outside of the work setting, such as life situations (Zheng et al., 2023). It is most readily applied to work-related experiences like thriving and engagement. Second, the mechanism of cross-domain resource transfer likely depends less on momentary, present-focused awareness and more on structural opportunities, familial support, or deliberate integration strategies. Although WCBA generates positive resources that can spill over into family life, workplace mindfulness itself might not inherently facilitate resource transfer across the work-family boundary. Finally, a broader measure of mindfulness might reveal a different moderating pattern than the work-contextualized measure.

### 5.1. Theoretical Contributions

This study’s theoretical contributions are centered around three main aspects.

Firstly, this study reconfirms the existence of a positive correlation between WCBA and employees’ work engagement, highlighting a potential beneficial aspect of WCBA. This responds to Park et al.’s (2020) call for research on the beneficial effects of connectivity behaviors. Previous research perspectives have often viewed WCBA as an “invisible exploitation” that undermines employee well-being, emphasizing its association with resource depletion and role conflict (He et al., 2023). However, by using intermittent time to acquire knowledge and integrate work and family resources for cross-domain competency development, employees can shift WCBA from an organizationally enforced temporal intrusion to an individually driven growth empowerment mechanism that significantly enhances work dynamics and engagement.

Secondly, this study explores the mechanism of action by which WCBA positively affects work engagement. Previous studies have mainly examined employees’ psychological experiences in a single domain (Yang et al., 2023; He et al., 2023). This study shifts the focus to the sustainable growth dynamics of employees and combines the work and family domains. In the pathway of thriving at work, WCBA may enhance employees’ flexibility and autonomy by providing informational, social, and instrumental support anytime and anywhere. This facilitates the satisfaction of their psychological needs, which in turn could promote cognitive flexibility and learning motivation. In the pathway of work-family enrichment, the findings indicate that WCBA may facilitate cross-domain resource augmentation through competence transfer and emotional spillover. Specifically, our study sample includes a relatively high proportion of participants with higher educational attainment, which may influence how individuals manage the boundaries between work and family. At the same time, the socio-cultural context in which the sample is situated generally emphasizes collective family interests and tends to regard work achievements as an important component of family well-being. Under this background, WCBA is more likely to be perceived by family members as a supportive action that contributes to the family’s long-term development. This provides a cognitive and socially supported basis for positive emotional spillover across domains. Furthermore, the discovery of the dual pathways innovatively integrates the JD-R model with the W-HR model. This integration not only confirms the mutual reinforcement between job resources and personal resources (Xanthopoulou et al., 2009) but also validates the feasibility of cross-domain resource transfer through the work-family enrichment pathway (Ten Brummelhuis & Bakker, 2012).

Thirdly, this study examines the moderating effect of workplace mindfulness on the mediation path where thriving at work links WCBA to work engagement. This study finds that employees with high workplace mindfulness are more effective at converting work connectivity behaviors into positive resources when engaging in work during non-work time. Specifically, employees with high workplace mindfulness avoid cognitive overload by focusing more on growth-oriented core tasks and ignoring low-value trivial information. Concurrently, their emotion regulation ability enables them to mitigate anxiety responses when confronted with work demands during non-work time, allowing them to approach tasks with a calm and composed mindset (Malinowski & Lim, 2015). This psychological regulation mechanism significantly amplifies the mediating effect of thriving at work. When employees maintain a high state of workplace mindfulness, improvisational learning triggered by WCBA is more likely to be systematically integrated, thereby fostering sustainable competence development. This finding not only responds to the academic focus on workplace mindfulness as a moderating variable (Shahbaz & Parker, 2021) but also extends its research scope from the traditional perspective of stress relief to the domain of resource conversion, thereby enriching the application scenarios of workplace mindfulness studies.

### 5.2. Practical Implications

This study offers several practical implications. First, organizations should adopt a nuanced and dynamic perspective on managing WCBA. Rather than implementing blanket prohibitions or unregulated expectations, managers can differentiate connectivity based on task urgency and job characteristics. Empowering employees with the autonomy to choose when and how to engage after hours can help transform fragmented connectivity into perceived resource gains, thereby mitigating its potential as a stressor and unlocking its motivational benefits. It is important to note that, without reasonable constraints, WCBA may evolve into an implicit expectation. Organizations should carefully manage the boundaries and intensity of such connectivity to prevent potential resource gains from being transformed into a counterproductive burden. Second, organizations can actively foster environments that support both pathways to engagement. To promote thriving, leaders can ensure that after-hours tasks are meaningful and aligned with employees’ growth goals and provide timely recognition for such efforts. To promote work-family enrichment, organizations can cultivate a family-supportive culture, offer resources for boundary management, and encourage leaders to model healthy work-life integration practices. This can help employees convert work-derived resources into family gains and complete the positive feedback loop back to work engagement. Third, organizations should invest in developing workplace mindfulness as a trainable personal resource. Through mindfulness training programs, employees can enhance their ability to regulate attention and emotion when engaging with work during non-work time. This can help them maximize the thriving benefits of WCBA while buffering against its depleting effects. Ultimately, in a hyper-connected era, building employees’ internal psychological resources may be more sustainable and effective than focusing solely on behavioral restrictions.

### 5.3. Limitations and Future Research Directions

While the findings of this study hold theoretical and practical value, certain limitations remain. First, the cross-sectional design has inherent limitations. Future research could adopt diary methods and integrate multi-source data to enhance the reliability of the conclusions. Second, this study focuses on resource migration at the individual level, but the diversification of family structures may alter the operational logic of work-family enrichment. For example, does the resource allocation conflict faced by single parents during non-work time weaken the resource gain effect? Future research could deeply analyze the nested effects of variables like generational differences, gender, and role expectations on the dual-path model. Third, workplace mindfulness did not moderate the association between WCBA and work-family enrichment in this study. The reasons and underlying mechanisms are worth exploring in future research. Concurrently, future research could incorporate contextual variables such as family-supportive leadership and individual differences in boundary management preferences or family role expectations to examine the boundary conditions of the enrichment pathway.

## 6. Conclusions

Guided by COR theory and integrating the JD-R model and the W-HR model, this study demonstrates a positive association between WCBA and work engagement and elucidates its underlying mechanisms. These findings indicate that WCBA can have a positive impact on work engagement by fostering employees’ thriving at work and their experience of work-family enrichment. Our study incorporates previously underexplored cross-domain resource dynamics, offering novel perspectives on WCBA. In addition, our study enriches the literature on workplace mindfulness by demonstrating its moderating role.

## Figures and Tables

**Table 1:** Results of confirmatory WCBA et al., 2011 factor analysis.

Model	*χ* ^2^	*df*	*χ* ^2^ */df*	RMSEA	CFI	TLI	SRMR
5-factor model (WCBA, TW, WFE, WM, WE)	2494.023	1585	1.574	0.042	0.905	0.901	0.051
4-factor model (WCBA, TW + WFE, WM, WE)	2620.244	1589	1.650	0.045	0.892	0.888	0.054
3-factor model (WCBA + TW + WFE, WM, WE)	3549.760	1592	2.230	0.061	0.796	0.788	0.074
2-factor model (WCBA + TW + WFE + WE, WM)	4087.153	1594	2.564	0.069	0.740	0.730	0.075
Single factor model (WCBA + TW + WFE + WM + WE)	4772.065	1595	2.992	0.078	0.669	0.657	0.084

Note: WCBA is work connectivity behavior after hours; TW is thriving at work; WFE is work-family enrichment; WM is workplace mindfulness; WE is work engagement; and + indicates factor merging.

**Table 2:** Results of descriptive statistics and correlation analysis.

Variables	1	2	3	4	5	6	7	8	9	10
1 Gender	1									
2 Age	−0.284 **	1								
3 Education	0.242 **	−0.093	1							
4 Marital status	0.310 **	−0.604 **	0.108	1						
5 Working tenure	−0.297 **	0.766 **	−0.112 *	−0.739 **	1					
6 WCBA	−0.120 *	0.092	−0.034	−0.121 *	0.096	1				
7 TW	−0.266 **	0.098	0.019	−0.075	0.006	0.259 **	1			
8 WFE	−0.183 **	0.172 **	0.061	−0.160 **	0.058	0.161 **	0.725 **	1		
9 WM	−0.110 *	0.121 *	0.017	−0.071	0.061	0.176 **	0.625 **	0.681 **	1	
10 WE	−0.312 **	0.253 **	0.043	−0.266 **	0.170 **	0.271 **	0.693 **	0.738 **	0.542 **	1
Mean	1.557	2.168	3.138	1.434	3.572	2.910	3.340	3.649	3.140	3.587
SD	0.498	0.817	0.711	0.496	1.414	0.894	0.491	0.707	0.810	0.540

Note: N = 327; * indicates *p* < 0.05, ** indicates *p* < 0.01.

**Table 3:** Results of hierarchical regression analyses.

Variables	1	2	3	4	5	6	7	8	9	10
Variables	TW			WFE			WE			
Gender	−0.290 ***	−0.269 ***	−0.216 ***	−0.170 **	−0.158 **	−0.103 *	−0.278 ***	−0.258 ***	−0.091 *	−0.154 ***
Age	0.179 *	0.173 *	0.074	0.268 ***	0.265 ***	0.160 **	0.238 **	0.233 **	0.125 *	0.057
Education	0.085	0.086	0.065	0.112 *	0.112 *	0.083 *	0.129 *	0.130 *	0.077 *	0.056
Marital status	−0.086	−0.066	−0.059	−0.208 **	−0.196 *	−0.188 **	−0.241 **	−0.222 **	−0.181 **	−0.092
Working tenure	−0.271 **	−0.267 **	−0.197 **	−0.339 ***	−0.337 ***	−0.267 ***	−0.259 **	−0.255 **	−0.089	−0.032
WCBA		0.231 ***	0.137 ***		0.130 *	0.026		0.221 ***	0.077 *	0.135 ***
TW									0.622 ***	
WFE										0.662 ***
WM			0.571 ***			0.647 ***				
WCBA * WM			0.082 *			0.020				
*R* ^2^	0.101	0.153	0.473	0.098	0.114	0.512	0.173	0.221	0.549	0.600
Δ*R* ^2^	0.101	0.052	0.007	0.098	0.017	0.000	0.173	0.048	0.328	0.388
*F*	7.219 ***	9.648 ***	35.645 ***	6.942 ***	6.876 ***	41.694 ***	13.420 ***	15.095 ***	55.408 ***	71.000 ***

Note: N = 327; * indicates *p* < 0.05, ** indicates *p* < 0.01, *** indicates *p* < 0.001.

**Table 4:** Results of path analysis.

Path	Effect	Boot SE	Boot 95% CI
Total effect: WCBA→WE	0.200	0.045	[0.111,0.289]
Direct effect: WCBA→WE	0.085	0.032	[0.023,0.147]
Indirect effect (Total)	0.115	0.037	[0.046,0.191]
Path 1: WCBA→TW→WE	0.061	0.021	[0.026,0.108]
Path 2: WCBA→WFE→WE	0.055	0.026	[0.008,0.109]

**Table 5:** Results of moderated mediation analysis.

Path	Workplace Mindfulness Level	Effect	Boot SE	Boot 95% CI
WCBA→TW→WE	Low (M – 1 SD)	0.035	0.040	[−0.043,0.119]
WCBA→TW→WE	High (M + 1 SD)	0.148	0.042	[0.073,0.237]
High-low difference		0.113	0.058	[0.004,0.234]

## Data Availability

The data that support the findings of the study are available from the corresponding author upon reasonable request.

## Abbreviations

The following abbreviations are used in this manuscript:

WCBA	Work Connectivity Behavior After-Hours
COR	Conservation of Resources
JD-R	Job Demands-Resources
W-HR	Work-Home Resources
